# Next-generation sequencing of FLT3 internal tandem duplications for minimal residual disease monitoring in acute myeloid leukemia

**DOI:** 10.18632/oncotarget.4333

**Published:** 2015-06-02

**Authors:** Jean-Emmanuel Bibault, Martin Figeac, Nathalie Hélevaut, Céline Rodriguez, Sabine Quief, Shéhérazade Sebda, Aline Renneville, Olivier Nibourel, Philippe Rousselot, Bérengère Gruson, Hervé Dombret, Sylvie Castaigne, Claude Preudhomme

**Affiliations:** ^1^ Laboratory of Hematology, CHRU de Lille, France; ^2^ Functional and Structural Genomic Platform, Lille, France; ^3^ University of Lille Nord de France, Lille, France; ^4^ Institut Pour la Recherche sur le Cancer de Lille, Lille, France; ^5^ Inserm, UMR-S1172, Lille, France; ^6^ Department of Hematology, Hôpital de Versailles, Le Chesnay, Université de Versailles-Saint Quentin, Versailles, France; ^7^ Department of Hematology, CHU d'Amiens, Amiens, France; ^8^ Department of Hematology, Hôpital Saint Louis, AP-HP, Paris, France

**Keywords:** acute myeloid leukemia, FLT3 internal tandem duplication, minimal residual disease, next-generation sequencing

## Abstract

Minimal Residual Disease (MRD) detection can be used for early intervention in relapse, risk stratification, and treatment guidance. FLT3 ITD is the most common mutation found in AML patients with normal karyotype. We evaluated the feasibility of NGS with high coverage (up to 2.4.10^6^ PE fragments) for MRD monitoring on FLT3 ITD. We sequenced 37 adult patients at diagnosis and various times of their disease (64 samples) and compared the results with FLT3 ITD ratios measured by fragment analysis. We found that NGS could detect variable insertion sites and lengths in a single test for several patients. We also showed mutational shifts between diagnosis and relapse, with the outgrowth of a clone at relapse different from that dominant at diagnosis. Since NGS is scalable, we were able to adapt sensitivity by increasing the number of reads obtained for follow-up samples, compared to diagnosis samples. This technique could be applied to detect biological relapse before its clinical consequences and to better tailor treatments through the use of FLT3 inhibitors. Larger cohorts should be assessed in order to validate this approach.

## INTRODUCTION

Acute myeloid leukaemia (AML) affects three to four individuals per 100 000 each year worldwide, making it is the commonest acute leukaemia in adults. The overall outcome is still poor, with 5-year overall survival below 50% [1]. Next-generation sequencing (NGS) methods have elucidated the pathogenic process, and enabled identification of mutations, some of which add prognostic information [2]. These new techniques have also unraveled new potential therapeutic targets, which are urgently needed. FLT3 is a class III family receptor tyrosine kinase acting as a cytokine receptor for FLT3 ligand. FLT3 mutations are among the most frequent mutations observed in AML. 20% of patients have Internal Tandem Duplications (ITD) of FLT3 and this rate increases to 28–34% of those with cytogenetically normal AML. FLT3 ITD predict poor outcome, especially when the ITD is located in the tyrosine kinase domain [3, 4]. The internal tandem duplications in FLT3 interferes with the auto-inhibitory function of the juxtamembrane domaine and constitutively activates the tyrosine kinase, leading to enhanced RAS, MAPK, and STAT5 signalling. Patients with FLT3 ITD have an increased risk of relapse, and shorter overall survival. Higher ratio of the mutated to wildtype allele is associated with worse outcome [5]. About one in ten patients with cytogenetically normal AML show mutations in the activation loop of the tyrosine kinase domain of FLT3, predominantly at codons 835 and 836, which also lead to constitutive tyrosine kinase activation. The influence on prognosis of this variant remains controversial [6, 7]. Several tyrosine kinase inhibitors are currently being investigated, since FLT3 could be a meaningful actionable therapeutic target AML. FLT3 ITD blasts are more susceptible to FLT3 inhibition than wild-type, a feature that was not found with the FLT3 tyrosine kinase domain mutation [8]. While first generation compounds such as midostaurin, lestaurtinib, sunitinib, and sorafenib have shown limited effect as single agents [9], the second-generation FLT3 inhibitors, such as quizartinib, show promising results [10]. Detecting patients with FLT3 ITD and being able to monitor minimal residual disease (MRD) on this marker could be an interesting approach in that regard. However, this technique is long, difficult, costly and doesn't allow for multiclonality detection during follow-up [11]. PCR and multiparametric flow cytometry (MPFC) have become the gold standard to monitor MRD because of their sensitivity and specificity [12]. Inconsistencies in MRD thresholds, uncertainty on the most informative MRD time points and the lack of standardized MRD assays have undermined its clinical utility. Clinical proof of improved outcome after MRD-driven therapy remains scarce. With its high sensitivity NGS could prove useful in detecting MRD and FLT3-ITDs are an interesting target in that regard. NGS could potentially detect mutational shifts between diagnosis and relapse, multiclonality at presentation, the outgrowth of a clone at relapse different from that dominant at diagnosis, variable insertion sites and lengths among patients [12]. The feasibility of FLT3 ITD MRD monitoring has been assessed by Thol et al with a limited coverage and sensitivity [13], but detecting FLT-ITDs can still be a challenge [14]. We present a method to monitor MRD on FLT3-ITD with a 10^6^ fold deep sequence coverage.

## RESULTS

### Sensitivity, scalability and comparison of the method to gold standard NGS ITD detection

**Figure 1:**
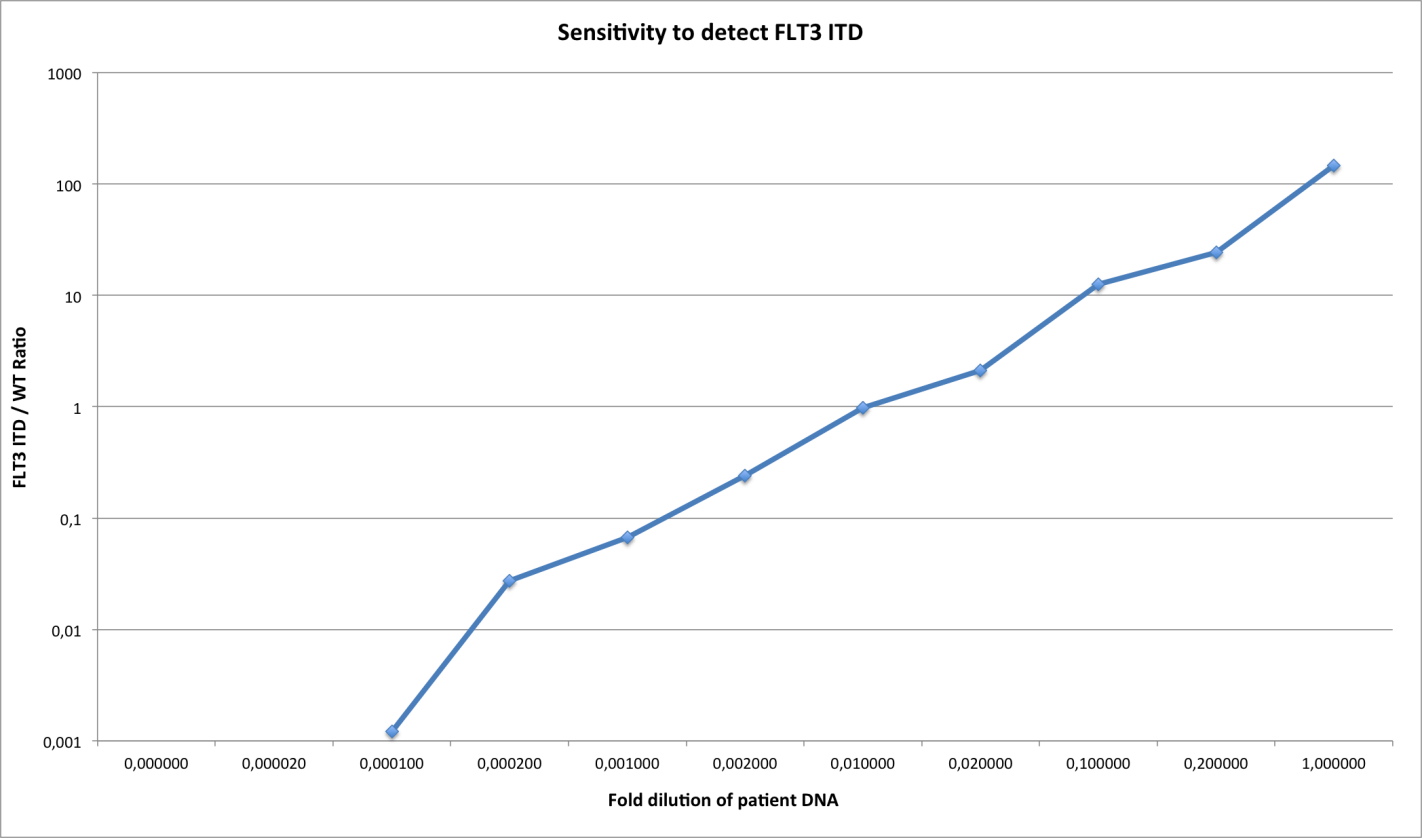
Sensitivity and scalability of the NGS doMreps FLT3 ITD detection

We initially sequenced 9 patients with HaloPlex Capture on a set of 38 genes including FLT3-ITD. All the ITD identified by fragment analysis (GeneScan^©^, Applied Biosystems, ThermoFisher Scientific, Waltham, Massachusetts, USA) were also identified by Pindel. With the aim of using NGS for MRD, we then tested if we could detect the FLT-ITD with a specific design, focusing on the FLT3-ITD locus followed by deep sequencing. To assess the feasibility of this method, 20 others patients were sequenced at diagnosis with this focused PCR. We also developed doMreps, a specific method to detect tandem duplications with high sensibility. Results for ITD detection were concordant for 17 patients (85%) between fragment analysis, NGS analysed with Pindel and NGS analysed with doMreps. In three samples Pindel and doMreps failed to detect the same ITD as fragment analysis. Results are shown in Table 1. Finally, we investigated the sensitivity to finish the feasibility study. Figure 1 shows the range and sensitivity of the detection method obtained from diluting a diagnosis positive sample in a negative control. The patient sequenced had a FLT3ITD/WT ratio x 100 of 119%. Our method was able to detect the 48 bp ITD after a 1/10.000 dilution.

**Table 1:** Comparison of methods for FLT3 ITD detection: enrichment with Haloplex or Focused PCR followed by fragment analysis (GeneScan), NGS with PINDEL or NGS with doMreps detection for patients with FLT3ITD positive AML at diagnosis. -: Missing values

UPN	Enrichment	FLT3-ITD size (GeneScan)	FLT3-ITD size (NGS with PINDEL)	FLT3-ITD size (NGS with doMreps)	FLT3ITD/WT Ratio
H1	Haloplex	39	39	NA	-
H2	Haloplex	30	30	NA	-
H3	Haloplex	48	48	NA	-
H4	Haloplex	27	27	NA	-
H5	Haloplex	69	69	NA	-
H6	Haloplex	72	72	NA	-
H7	Haloplex	24	24	NA	-
H8	Haloplex	18	18	NA	-
H9	Haloplex	27	27	NA	-
1	Focused PCR	36	36	36	0.85
2	Focused PCR	26	66	66	0.5
2	Focused PCR	18	19	18	2.7
4	Focused PCR	23	23	23	0.28
5	Focused PCR	21	21	21	0.45
6	Focused PCR	75	75	75	0.65
7	Focused PCR	51	51	51	0.9
8	Focused PCR	69	16	52	1.1
9	Focused PCR	21	64	23	0.2
10	Focused PCR	21	21	21	1.1
11	Focused PCR	45	45	45	1
12	Focused PCR	57	57	57	0.87
13	Focused PCR	26	26	26	-
14	Focused PCR	17	17	17	-
15	Focused PCR	38	38	38	-
16	Focused PCR	15	15	15	0.9
17	Focused PCR	63	63	63	1.06
18	Focused PCR	21	21	21	1
19	Focused PCR	39	21	43	3
20	Focused PCR	50	50	50	-

### NGS is concordant with fragment analysis with a higher sensitivity

**Figure 2:**
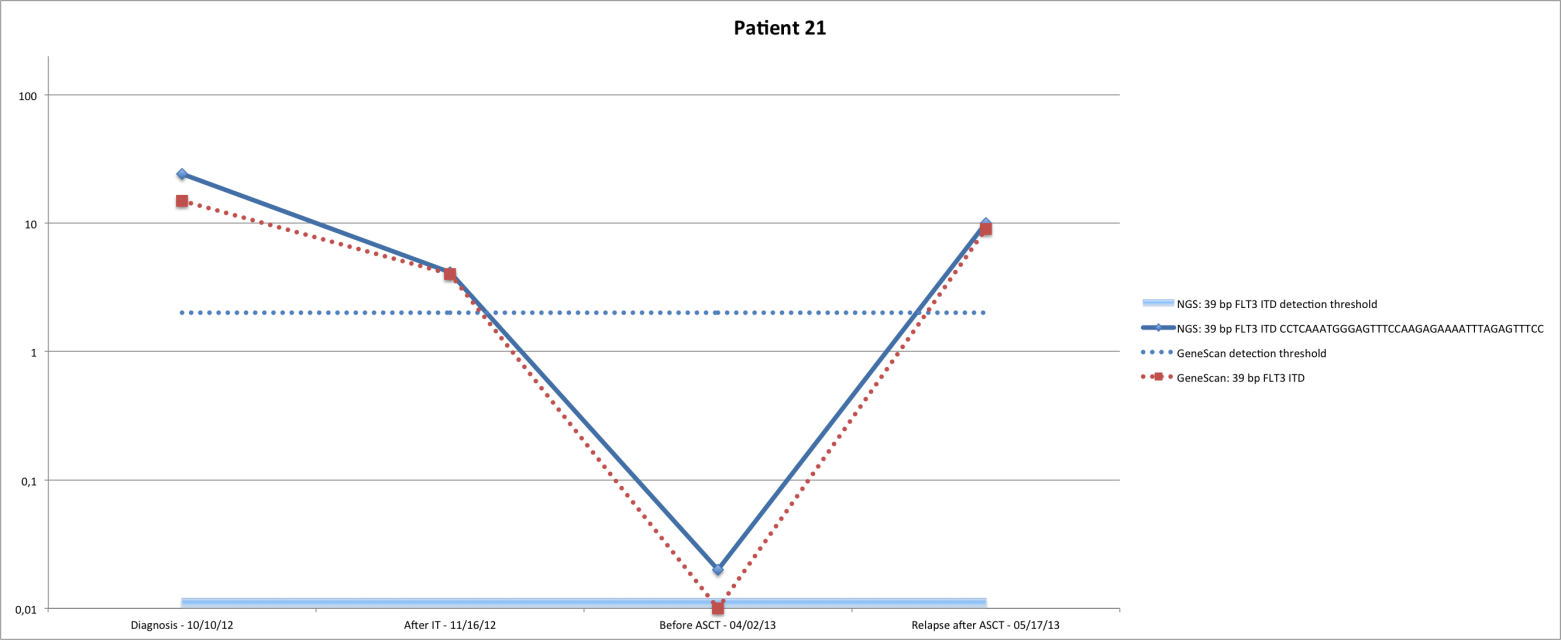

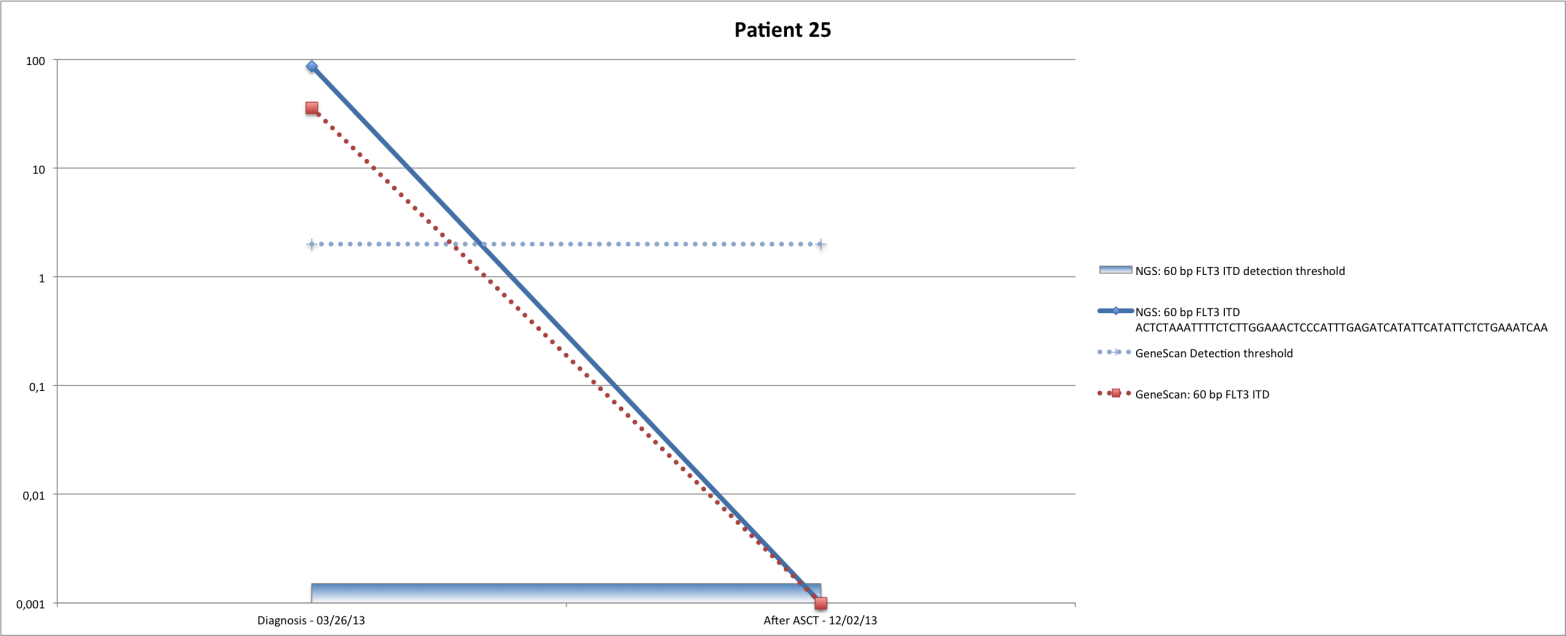

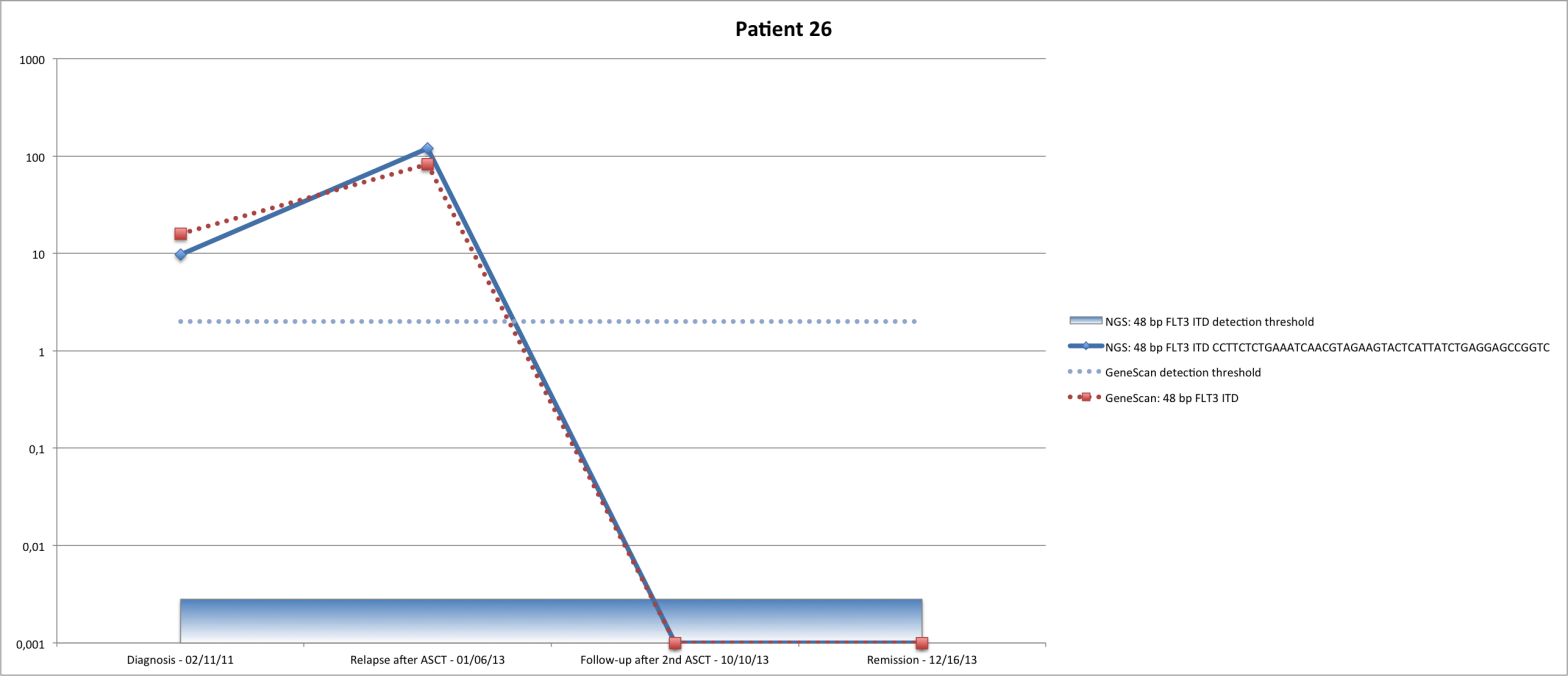

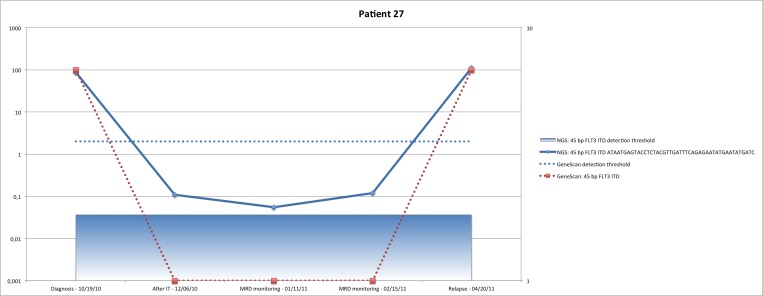

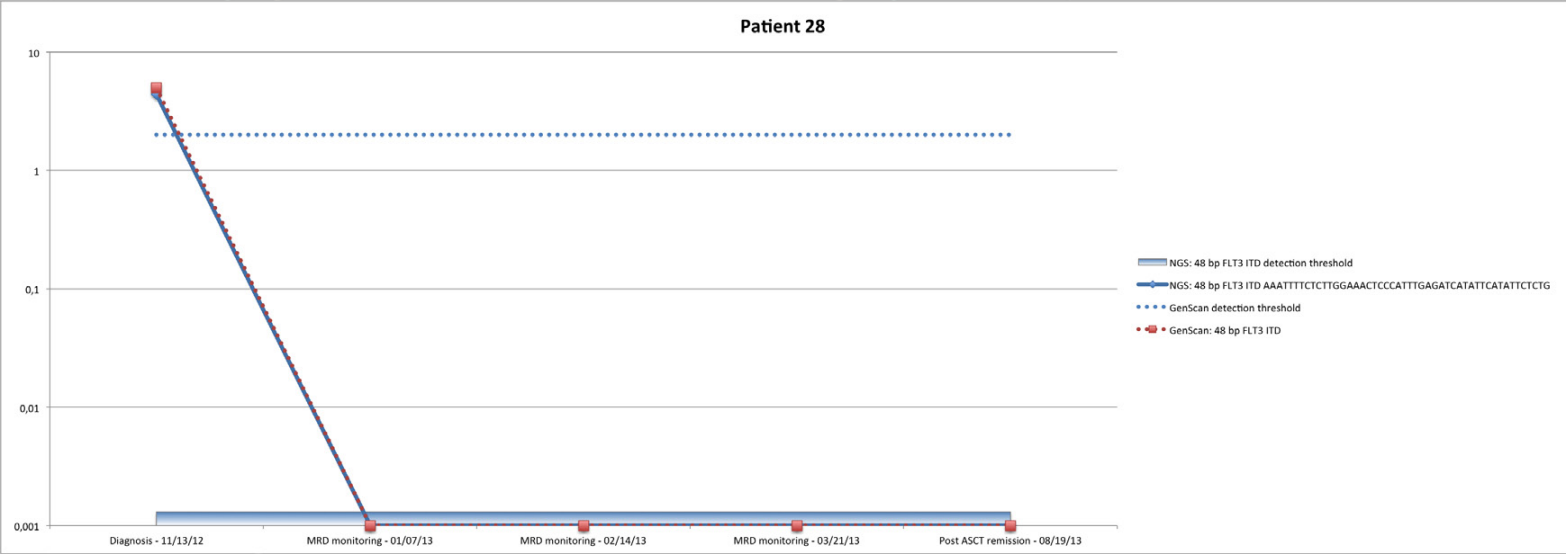
Concordant MRD monitoring with fragment analysis for FLT3 ITD and NGS for FLT3 ITD

In a second step, we sequenced 35 samples from 8 patients to assess the feasibility of our method for MRD monitoring. Clinical and biological characteristics of the studied patients are shown in Table 2. For five patients, NGS and fragment analysis found the same FLT3 ITD with the same length. The first of these patients (#21) was diagnosed with a FAB2 LAM in October 2012. A single 39 bp clone was found (Figure 2A). After IT (induction therapy), the patients still had 9% blasts and an ASCT (allogeneic hematopoietic stem cell transplantation) was performed in April 2013. MRD was positive before the treatment and the patient clinically relapsed in May 2013 with the same 39 bp clone, which was detected on both fragment analysis and NGS. All subsequent treatments failed and the patient died in October 2013. Patient #25 was diagnosed March 2013 (single 60 bp clone, Figure 2B) and treated according to protocol ALFA 1200, which failed. The patients received Azacitidine in June 2013 and an ASCT was done in August. The patient remained in remission as of January 2014 with a negative fragment analysis FLT3 ITD MRD and NGS MRD.

**Table 2:** Clinical and biological characteristics of the patients studied for MRD with NGS

UPN	Age / Gender	Initial FLT3-ITD size (qRT_PCR)	Initial FLT3-ITD size (NGS)	Initial qRT-PCR ratio (FLT3ITD/WT)	Initial NGS ratio (FLT3ITD/WT)	WBC Count (G/l)	FAB subtype	Response to IT	ASCT	Relapse	Death
21	68/M	39	39	15	24	67870	AML2	Failure	Yes	Yes	Yes
22	54/F	39	39	-	-	10670	AML5B	CR	No		Yes
		-	60	-	-						
23	34/F	-	30	-	0.82	NA	NA	CR	No	Yes	Yes
		75	75	86	0.9						
24	53/F	51	51	60	56.4	411000	AML1	CR	Yes	Yes	Yes
			84	-	0.68						
25	61/F	60	60	36	86.7	229950	NA	Failure	Yes	No	No
26	34/M	48	48	16	9,7	30000	AML4	CR	Yes	Yes	No
27	40/M	45	45	100	86.1	325000	AML5A	CR	No	Yes	Yes
28	62/M	48	48	5	4.4	196000	AML1	CR	Yes	No	No
		183	-								

Abbreviations: UPN, unique patient number; CR, complete remission; FAB, French–American–British; ASCT, allogeneic hematopoietic stem cell transplantation; NA, not available; MUT, mutated; WBC, white blood cell; IT, Induction therapy ; WT, wild-type.

Patient #26 was diagnosed with FAB4 LAM in February 2011, with a single 48 bp clone detected (Figure 2C). He was in CR (complete response) after induction therapy and received an ASCT in July 2011. The patient had a first relapse in April 2012. A second induction therapy was performed and the patient was once again in CR. The patient had a second relapse in January 2013, as shown by the rise of the same 48 bp clone and a second ASCT was done in September 2013. The two follow-up samples (October and December 2013) were negative by NGS and fragment analysis. The patient is since still in remission (September 2014). Patient #27 was diagnosed with FAB5a LAM in October 2010 (single 45 bp clone, Figure 2D) and treated in the ALFA 07-02 protocol. He was then in CR, with a negative fragment analysis MRD but a positive NGS MRD in three different samples. He relapsed in April 2011 and salvage chemotherapy with Amsacrine and Aracytine was given. After a second treatment failure, he was included in the AC220 protocol in June 2011 with a partial response. Three months later an ASCT was performed and failed (20% blasts). He died in December 2011. Patient #28 was diagnosed with FAB1 LAM in November 2012. Fragment analysis detected two clones of 45 and 183 length, while NGS detected only one of 45 bp (Figure 2E). He received induction therapy and was in CR. In June 2013, he relapsed and an ASCT was performed in July. The patient has been in remission ever since (July 2014), with a negative NGS MRD. NGS did not detect the 183 bp clone because it had been fragmented inside the ITD during the “tagmentation” required for the library preparation before sequencing. As we need to sequence a full ITD to efficiently detect and quantify an ITD, our protocol wasn't able to detect it.

### NGS shows multiclonality and mutational shifts between diagnosis and relapse

**Figure 3:**
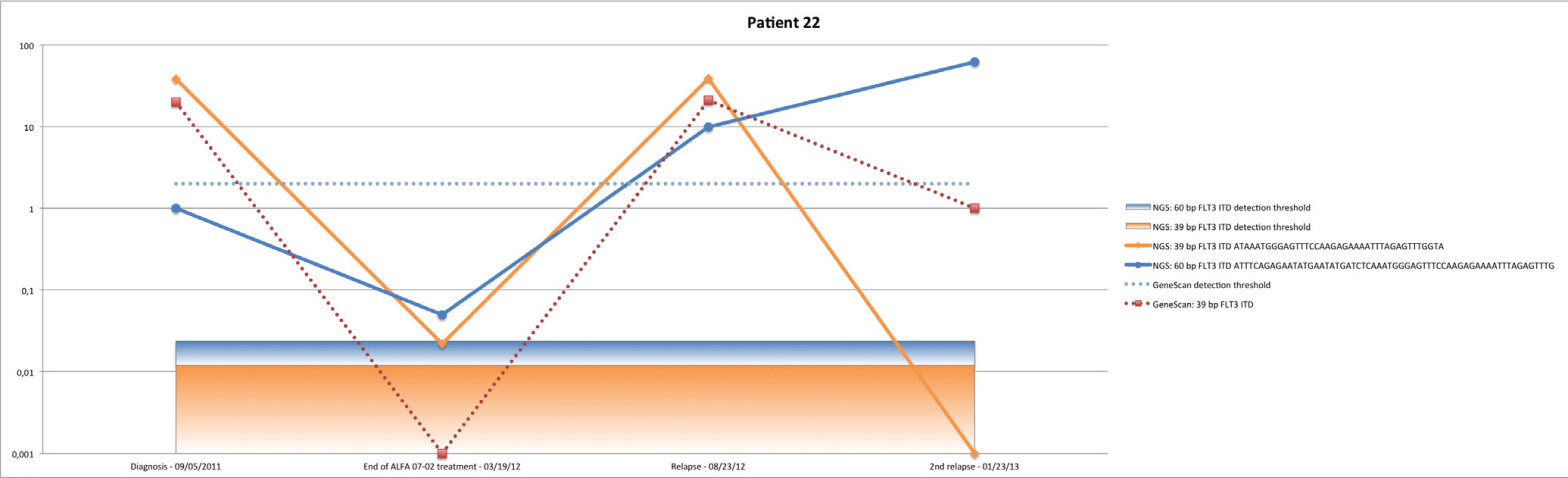

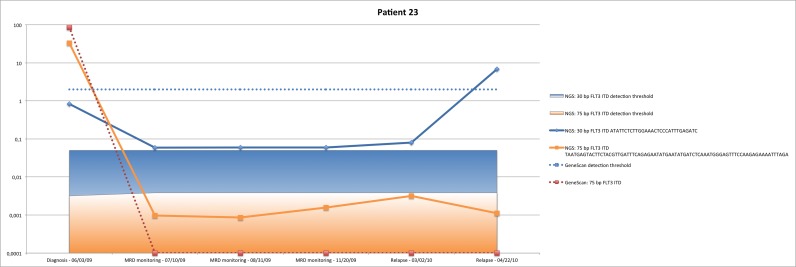

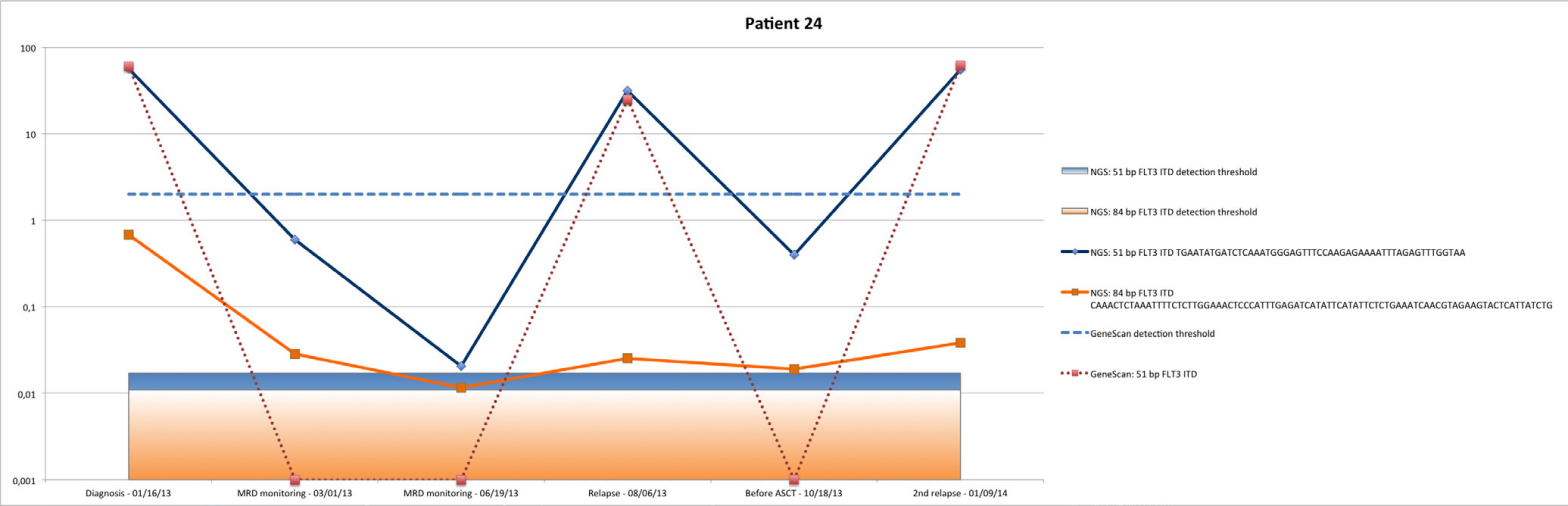
NGS detects polyclonality in patient #22 (A), patient #23 (B) and patient #24 (C) Mutational shifts can be seen for patient #22 **A.** and #23 **B.**.

For three patients, NGS was able to detect two clones instead of one. In these patients, we found a mutational shift between diagnosis and relapse. Patient #22 was diagnosed with FAB5b LAM in September 2011. Fragment analysis found a single 39 bp clone, while NGS found two clones: one with a 39 bp length (dominant) and a second with a 60 bp length (Figure 3A). The patient was treated in the ALFA 07-02 protocol and was in CR. At this time (March 2012), fragment analysis was negative while NGS was positive for the two clones. In August 2012, he relapsed with the two clones, as detected by NGS. He was treated with salvage chemotherapy and was once again in CR. In January 2013, a second relapse was found, but NGS could not detect the initial dominant clone and was only able to detect the 60 bp clone. The patient received AC220 in February 2013 and died the same month of febrile neutropenia. Patient #23 was diagnosed in June 2009. Fragment analysis found a single 75 bp clone, but NGS detected two clones: a dominant 75 bp clone and a second 30 bp clone (Figure 3B). The patient received IT according to ALFA 07-02 protocol. He was in CR and MRD negative by NGS and fragment analysis for the major clone, but positive for the minor clone. He relapsed in March 2010 with only the minor clone identified at diagnosis, responsible for the relapse. Salvage chemotherapy failed and the patient died in August 2010. Patient #24 was diagnosed in January 2013 with FAB1 AML. Fragment analysis detected a 51 bp clone, while NGS found the same dominant clone with a second 84 bp clone (Figure 3C). The patient was in CR after IT, but NGS MRD remained positive. The patient relapsed with the same profile as diagnosis. He was again in CR after salvage chemotherapy but the NGS clones remained positive before the ASCT performed in October 2013. He relapsed in February 2014, also with the same NGS profile and was included in a phase 1 trial. He died in May 2014.

## DISCUSSION

Before next-generation sequencing, two groups of mutations were recognized. Class I mutations constitutively activate signalling pathways, driving proliferation and uncontrolled growth of progenitors. Class II mutations affect transcription factors, leading to impaired haemopoietic differentiation and dysregulated self-renewal capacities. Class I and II mutations can occur together in AML. Acquisition of genetic changes gives rise to clonal heterogeneity with a subclonal architecture. High clonal diversity is associated with adverse outcome in AML probably because of an increased risk of a subclone acquiring resistance to therapy [15]. AML subclones can be detected, quantified, and followed by our method. AML relapse emerges from incompletely eradicated founder clones, rather than from development of new malignant clones [16, 17]. MRD detection is relevant for early intervention in relapse, risk stratification, and treatment guidance. Patients are classified as positive MRD (above a given threshold) or negative MRD (below a given threshold), but this does not translate the biological reality. The chosen threshold determines the sensitivity and specificity of an MRD assay [18]. But these thresholds are highly dependent on methodology and the best method to use is still a matter of debate.

While PCR is currently the gold standard for MRD monitoring, it is still difficult to use for FLT3 ITD monitoring [12]. NGS, with its increased sensitivity, might be useful for MRD targets like FLT3-ITD. Two studies have assessed the feasibility of using NGS to monitor MRD on FLT3 ITD in AML patients. In their study, Thol et al used Roche's 454 Sequencer to analyze five pediatric and five adult patients with a diagnosis of AML and FLT3-ITD at diagnosis and during follow-up [13]. In total, 2,563,550 sequencing reads were generated. Thirty-five samples from 10 patients (five adults and five children) with FLT3-ITD were sequenced with an average read depth of 15,278 reads per sample (range 5,525–24,997 reads). The sensitivity of NGS to detect mutated alleles was assessed by sequencing serial dilutions of a patient sample with a known FLT3 ITD/wild type ratio at diagnosis. They calculated the theoretical level of detection to be 1 in 4,630 sequences but their serial dilution demonstrated a linear decrease in the allelic ratio down to the 5×10-4 dilution. The author explain their decision to cover x10,000 sequences per amplicon was made to limit the costs of the analysis. They also pointed out that the number of expected reads (and thus sensitivity) is scalable. They concluded that, as costs of NGS decline, NGS may become an attractive tool to assess MRD in the future, especially in patients treated with FLT3 inhibitors. The high sensitivity of our method seems to confirm these results, since we detected clones at diagnosis at a very low level that later emerged at relapse. More recently, Spencer et al. assessed several different bioinformatics methods to monitor MRD on FLT3 [14]. They used a multigene, targeted NGS assay to obtain deep sequence coverage (>1000-fold) of FLT3 and 26 other genes from 22 FLT3 ITD-positive and 29 ITD-negative specimens to examine the performance of several commonly used NGS analysis tools for identifying FLT3 ITD mutations. FLT3 sequencing was performed via targeted next-generation sequencing on a HiSeq 2000 sequencing system (Illumina), using a next-generation sequencing-based panel (WUCaMP27; Washington University Genomic Pathology Services, St. Louis, MO) for detecting somatic mutations in FLT3 and in 26 other genes that are frequently mutated in cancer. They compared the ability of several software tools to detect and quantify FLT3 ITD and concluded that using NGS to detect FLT3 ITD in an integrated manner with several other mutations was feasible and that PINDEL was the best tool to do so. Our method proved as accurate as PINDEL, while being much faster and lighter, since we did not require to realign the sequences (which is very time-consuming). Our method can be directly performed on the FASTQ files that the sequencer outputs.

Our study demonstrates that the insertion site, insertion length, number of individual clones, and allelic ratio of FLT3 insertions can be detected by NGS in a single analysis with a high sensitivity. Using NGS, we were able to assess clonal dominance over the course of the disease. MRD assessment in the majority of AML patients could be performed with this technique, associated with a panel of the most commonly found mutations. Individual primer/probe designs for MRD studies are also no longer required with NGS. NGS is a scalable tool, meaning that by changing the read count, we can change the sensitivity of MRD monitoring. High-sensitivity could detect molecular aberrations at presentation and during MRD monitoring in more patients, making them eligible for targeted therapy. Several classes of FLT3 inhibitors are in development with varying degrees of potency and selectivity for the target [10]. These agents are generally well tolerated compared with traditional cytotoxic agents, unfortunately, like many tyrosine kinase inhibitors, their single-agent clinical activity has been modest and major clinical responses in AML patients receiving single-agent FLT3 inhibitors have been rare. However, with a correct MRD monitoring, ways to optimize the timing of their prescription could be improved.

## CONCLUSION

Next-generation sequencing with a high coverage offers an efficient way to monitor MRD on FLT3 ITD for AML patients. It offers a higher sensitivity than PCR and the ability to detect mutational shifts between diagnosis and relapse, variable insertion sites and lengths among patients. It could potentially be used to better tailor treatments, through the timely use of FLT3 tyrosine kinase inhibitors at the biological relapse, as detected by NGS, before the clinical relapse.

## MATERIALS AND METHODS

### Patients and treatment

Thirty seven adult patients with a diagnosis of AML with normal karyotype and positive for FLT3-ITD were analyzed by NGS at diagnosis and during follow-up. Collection of bone marrow (BM) and peripheral blood (PB) samples were performed at AML diagnosis, after induction therapy, after relapse and after each consolidation courses.

### HaloPlex enrichment and sequencing

In a first step, we analyzed 9 diagnosis samples processed with our standard NGS diagnosis routine protocol. We used Haloplex following Agilent standard recommendations on a custom design targeting a set of 44 genes. The samples were sequenced in batches of 10 on a MiSeq Illumina instrument with 2×150bp chemistry.

### FLT3-ITD amplicons (focused protocol)

In a second step, we sequenced twenty eight patients, at diagnosis and during follow-up, with our FLT3-ITD focused protocol. The following FLT3 primers were used: Forward: 5′ FAM_GCA-ATT-TAG-GTA-TGA-AAG-CCA-GC_3′; Reverse : 5′-CTT-TCA-GCA-TTT-TGA-CGG-CAA-CC-3′. FLT3 was amplified with The Fast Start High Fidelity PCR system^©^ (Roche Life Science, Basel, Switzerland). Conditions for amplification were as follow: denaturation at 95° C for 10 min, followed by 10 cycles of 95°C for 30 sec, 63°C for 30 seconds, and 72°C for 30 seconds, followed by 20 cycles of 95°C for 30 sec, 58°C for 30 seconds, and 72°C for 30 seconds with final extension at 72°C for 10 min. PCR products were purified with the Agencourt Ampure XP^©^ kit (Beckman Coulter, Pasadena, California, USA). DNA concentration of the purified amplicons was quantified with the Quant-iT PicoGreen dsDNA Assay^©^ (Life Technologies, Thermo Fisher Scientific, Waltham, Massachusetts, USA).

### DNA sequencing for MRD monitoring and diagnosis

Library was prepared from FLT3 ITD amplicons (0.2 ng/ml) using the Nextera XT^©^ kit according to the manufacturer's recommendations (Illumina, San Diego, California, USA). DNA was simultaneously fragmented and tagged (“tagment”) every 150 bp, adding the adapter sequences in the process.[19] A limited-cycle PCR reaction used these adapter sequences to amplify the insert DNA. Index sequences on both ends of the DNA were also added during the PCR reaction, to enable dual-indexed sequencing of pooled libraries. Indexed libraries were purified with the Agencourt Ampure XP^©^ kit and equal volumes of normalized library were combined and diluted in hybridization buffer in preparation for cluster generation and sequencing. Samples were sequenced on a MiSeq^©^ (Illumina, San Diego, California, USA) in 5 runs (with 3 to 12 samples per run). Sixty uL of 12.5 pM PhiX were mixed with samples for each run. To avoid contamination between diagnosis and follow-up samples from the same patients, we pooled samples to sequence all diagnosis together (in 2 runs) and mixing all follow-up samples from all patients (in the 3 remaining runs).

### Bioinformatics and statistical analysis

The quality of the FASTQ files was controlled with FASTQC [20]. Pindel analyses were done after a standard BWA alignment [21, 22]. Raw results from Pindel were exported to VCF format and then manually reviewed. For our analysis method called doMreps, the demultiplexed paired-end FASTQ files were imported from the MiSeq and merged as single-end reads using the PEAR software [23]. A slightly modified version of mReps (only output format was modified for an easier parsing) was used to identify the FLT3 ITD sequence.[24] doMreps is available online for Linux and Mac OSX/Darwin: https://github.com/mafouille/doMreps

Once the sequence had been identified, we used a custom PERL script to count the occurrence of the specific breakpoint in all samples and all runs and calculate the positivity threshold for each sample in its own run using a Fisher Exact test with R [25]. Sensibility was also computed as the minimal FLT3-ITD ratio, based on each sample depth of coverage, to have a Fisher Exact Positive test. The Fisher Exact Test compares the number of overall read for the sample, to the reads in control samples (other patients). This final step allows us to control potential contamination problems. FLT3 ITD ratio was computed as the number of read with the FLT3 ITD breakpoint divided by the number of reads with the FLT3 WT breakpoint.

In parallel, initial screening for FLT3-ITD were carried out as previously reported [11]. The mutant FLT3/wild-type allelic ratio found at diagnosis was considered to be the baseline value of each patient. FLT3-ITD levels at different follow-up time-points were assessed by fragment analysis and expressed as the decimal log reduction in respect to the baseline value of each patient. NGS results were then reported and correlated for each patient.
